# Evidence for Sigma Factor Competition in the Regulation of Alginate Production by *Pseudomonas aeruginosa*

**DOI:** 10.1371/journal.pone.0072329

**Published:** 2013-08-22

**Authors:** Yeshi Yin, T. Ryan Withers, Xin Wang, Hongwei D. Yu

**Affiliations:** 1 Department of Biochemistry and Microbiology, Joan C. Edwards School of Medicine at Marshall University, Huntington, West Virginia, United States of America; 2 Department of Pediatrics, Joan C. Edwards School of Medicine at Marshall University, Huntington, West Virginia, United States of America; 3 Institute of Plant Protection and Microbiology, Zhejiang Academy of Agricultural Sciences, Hangzhou, China; 4 Progenesis Technologies, LLC, Huntington, West Virginia, United States of America; Tulane University, United States of America

## Abstract

Alginate overproduction, or mucoidy, plays an important role in the pathogenesis of *P. aeruginosa* lung infection in cystic fibrosis (CF). Mucoid strains with *mucA* mutations predominantly populate in chronically-infected patients. However, the mucoid strains can revert to nonmucoidy *in vitro* through suppressor mutations. We screened a mariner transposon library using CF149, a non-mucoid clinical isolate with a misssense mutation in *algU* (AlgU^A61V^). The wild type AlgU is a stress-related sigma factor that activates transcription of alginate biosynthesis. Three mucoid mutants were identified with transposon insertions that caused 1) an overexpression of AlgU^A61V^, 2) an overexpression of the stringent starvation protein A (SspA), and 3) a reduced expression of the major sigma factor RpoD (σ^70^). Induction of AlgU^A61V^ *in trans* caused conversion to mucoidy in CF149 and PAO1*DalgU*, suggesting that AlgU^A61V^ is functional in activating alginate production. Furthermore, the level of AlgU^A61V^ was increased in all three mutants relative to CF149. However, compared to the wild type AlgU, AlgU^A61V^ had a reduced activity in promoting alginate production in PAO1*ΔalgU*. SspA and three other anti-σ^70^ orthologues, *P. aeruginosa* AlgQ, *E. coli* Rsd, and T4 phage AsiA, all induced mucoidy, suggesting that reducing activity of RpoD is linked to mucoid conversion in CF149. Conversely, RpoD overexpression resulted in suppression of mucoidy in all mucoid strains tested, indicating that sigma factor competition can regulate mucoidy. Additionally, an RpoD-dependent promoter (*P_ssrA_*) was more active in non-mucoid strains than in isogenic mucoid variants. Altogether, our results indicate that the anti-σ^70^ factors can induce conversion to mucoidy in *P. aeruginosa* CF149 with *algU*-suppressor mutation via modulation of RpoD.

## Introduction

The Gram-negative bacterium *P. aeruginosa* is an important opportunistic pathogen in humans, and has the potential to proliferate in a wide range of niches. *P. aeruginosa* is one of the major etiological agents of hospital-acquired infections and ventilator-associated pneumonia [1]. More importantly, *P. aeruginosa* is the leading cause of morbidity and mortality in cystic fibrosis (CF) patients [2].

*P. aeruginosa* can produce a capsule-like polysaccharide called alginate. Overproduction of alginate is also known as mucoidy [3]. Mucoid conversion facilitates the establishment of persistent infection with *P. aeruginosa* in CF. The role of alginate in pathogenesis includes: increased resistance to antibiotics [2], increased resistance to phagocytic killing [4], [5] and evasion of the host’s immune response [4]. However, the mucoid phenotype observed in CF isolates is extremely unstable *ex vivo* [6], [7], [8]. Reversion to non-mucoidy is common *in vitro* in the absence of a selective pressure, and *in vivo* during the end-stage of CF disease [9]. Although, environmental signals such as high osmolarity, nitrogen or phosphate starvation, and ethanol-induced membrane perturbation can activate transcription of *algD* encoding the key enzyme for alginate biosynthesis [10], the selective pressure for mucoid conversion of *P. aeruginosa* in CF respiratory environment is not fully understood.

Several genes in *P. aeruginosa* are known to regulate alginate production. Specifically, AlgU (AlgT, σ^22^) is an alternative sigma factor that drives the transcription of *algD* [11]; MucA is a trans-membrane protein that negatively regulates alginate production by sequestering AlgU [12]; MucB and proteases AlgW, MucP and ClpXP affect alginate production by altering the stability of MucA [13]. Mutations in *mucA* are recognized as the primary reason for mucoid conversion in CF isolates [14], [15], [16], [17]. However, the reversion from a mucoid to a non-mucoid phenotype is still possible. Sautter *et al.* isolated 34 spontaneous non-mucoid variants from a MucA truncated mucoid stain PDO300 [18]. In another study, 70% of the non-mucoid CF isolates carried a *mucA* mutation [19].

The aim of this study was to better understand the alginate regulation by determining if there are upstream mutations that restore alginate overproduction to a clinical nonmucoid, *algU mucA* double mutant (CF149). To achieve this objective, we conjugated the mariner transposon plasmid pFAC [20] into the *mucA* mutant CF149 with an alginate-suppressing mutation [21], and screened for mucoid variants. Three genes were identified that regulated mucoidy in CF149. Mechanistic studies suggest that mucoid conversion in MucA truncated strains including CF149, is related to competition between the two sigma factors, the major house keeping sigma factor RpoD and AlgU for binding to the core RNA polymerase (RNAP). Additionally, we documented that anti-σ^70^ factors have the ability to induce mucoidy in the suppressed non-mucoid *P. aeruginosa* strain CF149.

## Materials and Methods

### Bacteria Strains, Plasmids, and Growth Conditions

Bacterial strains and plasmids used in this study are shown in Table S1. *Escherichia coli* strains were grown at 37°C in Lennox broth (LB) or LB agar. *P. aeruginosa* strains were grown at 37°C in LB or on *Pseudomonas* isolation agar (PIA) plates (Difco). When required, carbenicillin, tetracycline or gentamicin were added to the broth or plates. The concentrations of carbenicillin, tetracycline or gentamicin added in LB broth or plates were 100 µg ml^−1^, 20 µg ml^−1^ and 15 µg ml^−1^, respectively. The concentration of these antibiotics added to PIA plates were 300 µg ml^−1^, 150 µg ml^−1^ or 300 µg ml^−1^, respectively.

### Phage Culture and Genomic DNA Extraction

*E. coli* BB was inoculated into 6 ml LB, and incubated at 37°C with shaking. At an OD_600_ of 0.6, 200 µl of T4 phage stock was added to the culture, and incubated overnight. Phage lysates were collected using a 0.2 µm filter. Phage genomic DNA was extracted using the standard procedure with phenol:chloroform:isoamyl alcohol, and precipitated with ethanol.

### Transformation and Conjugation

The One Shot® TOP10 (Invitrogen) chemical transformation method was used according to the supplier’s instruction. The transfer of plasmids from *E. coli* to *Pseudomonas* was performed via triparental conjugations using the helper plasmid pRK2013 [22].

### Transposon Mutagenesis

Biparental conjugations were carried out for transposon mutagenesis, using *E. coli* SM10 λpir carrying plasmid pFAC as the donor strain [20] and CF149 as the recipient strain. After incubation, bacteria were collected and streaked onto PIA plates supplemented with gentamicin (300 µg ml^−1^). Mucoid colonies were identified and subjected to further genetic analyses. The chromosomal DNA of mucoid mutants was isolated using the QIAamp genomic DNA Extraction kit (Qiagen). Approximately, 2 µg DNA was digested with *SalI* overnight at 37°C followed by purification and self-ligation using Fast-Link DNA ligase (Epicentre). The circular closed DNA was used as template for inverse PCR using GM3OUT and GM5OUT primers [23]. The PCR products were purified and sequenced. Finally, southern blot hybridization was used to monitor the copy number of transposon insertions using the Gm^R^ gene as the probe [24].

### Protein Preparation, SDS-PAGE and Western Blotting

Bacteria were cultured on PIA plates for 24 hrs and then collected for cell lysis. Following sonication, the protein concentration of the resulting supernatant was measured using the Bio-Rad Dc protein assay reagents (Bio-Rad). Equal amounts protein were mixed with 2×sample loading buffer and separated on a pre-cast SDS-PAGE gels (Bio-Rad); Total proteins were transferred to PVDF membrane (GE) for immuno-detection. A primary monoclonal antibody of rat anti-HA (Roche) was used at a dilution of 1∶5000, while a goat anti-rat immunoglobulin G (heavy and light chains) conjugated with horseradish peroxidase (Pierce) (1∶5000) was used as the secondary antibody. The immunoreactive proteins were visualized using the Amersham ECL kit (GE).

### Alginate Assay

*P. aeruginosa* strains were grown at 37°C on triplicate PIA plates for 24 hrs. The bacteria were collected and suspended in PBS. The OD_600_ of bacterial suspension in PBS, which corresponds to the bacterial density, was measured. The amount of uronic acid was analyzed in comparison with a standard curve made with D-mannuronic acid lactone (Sigma-Aldrich), as previously described [25].

### β-galactosidase Activity Assay

*Pseudomonas* strains carrying the plasmid pLP170 containing the P*_ssrA_*, P*_algD_* and P*_algW_* promoters fused with the promoterless *lacZ* were cultured on three PIA plates. After 24 hrs, bacterial cells were harvested and re-suspended in PBS. OD_600_ was measured and adjusted to approximately 0.3. Cells were then permeabilized using toluene, and the β-galactosidase activity was measured at OD_420_ and OD_550_. The results of Miller Units were calculated according to this formula: Miller Units = 1000×[OD_420_–(1.75×OD_550_)]/[Reaction time (minutes)×Volume (ml)×OD_600_] [26]. The reported values represent an average of three independent experiments with standard error.

### RNA Isolation and Real-time PCR

Bacteria total RNA were extracted with a RNeasy Mini Kit (QIAGEN, USA) according to the manufacturer’s instructions. The real-time PCR assays were performed on ABI PRISM® 7000 (ABI, USA) with One Step SYBR® PrimeScript™ RT-PCR KIT II (TaKaRa, Japan) according to the manufacturer’s instructions. The *rpoD* gene was amplified using primers *rpoD*-RT-F (5′-AGA AGG ACG ACG AGG AAG A-3′) and *rpoD*-RT-R (5′-GCA CCA GCT TGA TCG GCA TGA -3′). The 16S rRNA gene was amplified using primers UniF340 (5′-ACT CCT ACG GGG AGG CAG CAG T-3′) and UniR514 (5′-ATT ACC GCG GCT GCT GGC-3′) [27]. The relative expression level of *rpoD* was normalized to 16S rRNA, and calculated according to the formula: fold change = 2^−ΔΔct^ [28].

### Measurement of Bacterial Growth

The *Pseudomonas* strains were grown in LB culture medium overnight at 37°C, then diluted to OD_600_ = 0.5 using PBS. Then 1 ml bacteria suspension was inoculated into a 250 ml flask containing 50 ml *Pseudomonas* Isolation Broth (PIB; Alpha Biosciences). To measure bacterial growth, OD_600_ was monitored every 2 hrs for 24 hrs. Triplicate of samples at each time point were measured, and the means with standard error were used to generate the growth curve.

### Statistical Analysis

The student *t* test and one-way ANOVA were performed using the statistical software SPSS 13.0 (IBM, US) with P<0.05 considered as significant.

## Results

### Identification of Alginate Regulators in a *P. aeruginosa* Strain with a Suppressor Mutation

**Figure 1:**
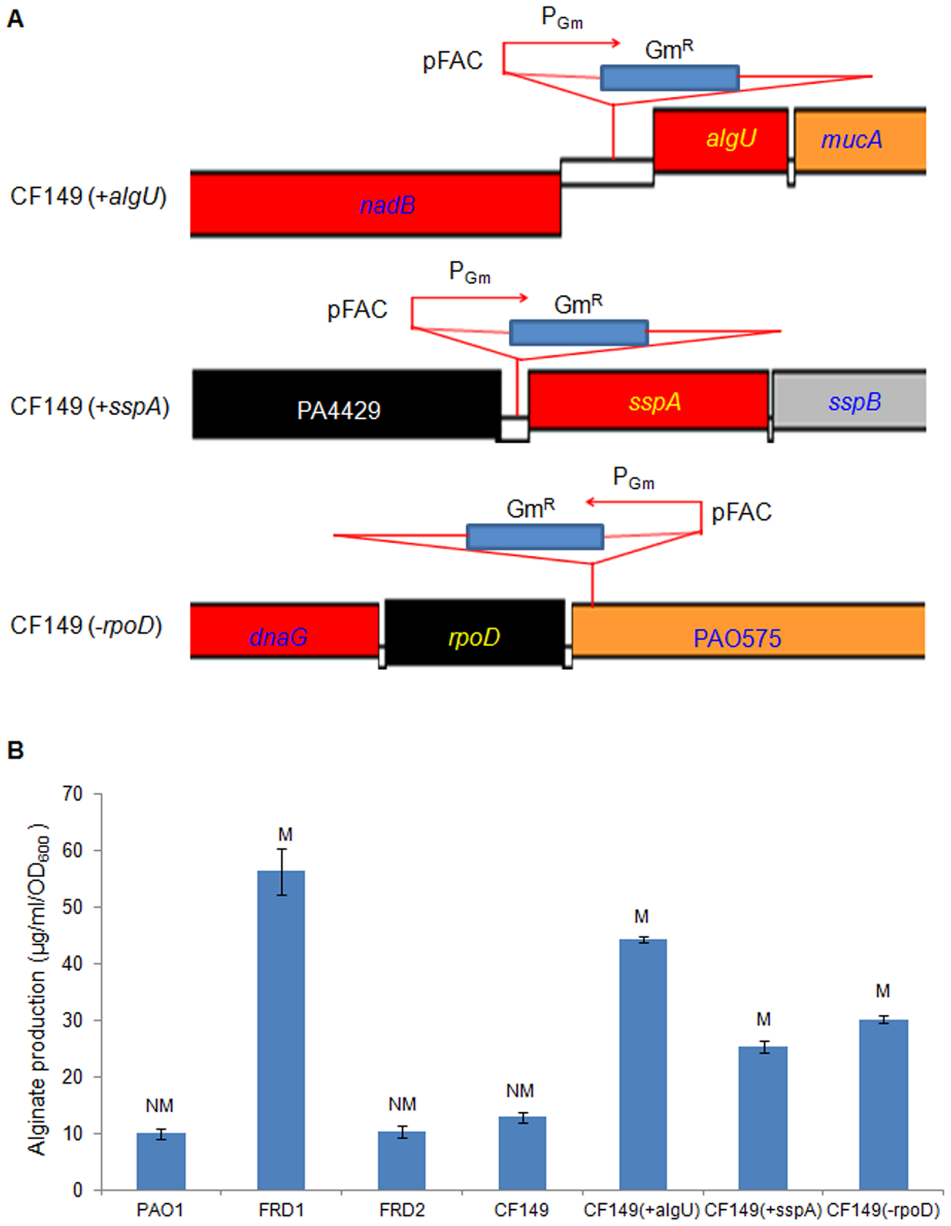
Increased alginate production in three mutants of CF149 with an *algU*-suppressor mutation. (A) Schematic diagram showing the transposon insertions of CF149 (+*algU*), CF149 (+*sspA*), and CF149 (*−rpoD*), respectively. (B) Alginate production of CF149 (+*algU*), CF149 (+*sspA*), and CF149 (*−rpoD*) in comparison to other strains of *P. aeruginosa*. Three mucoid mutants were identified as a result of a transposon library screen. Alginate production was measured on PIA plates after incubation at 37°C for 24 hrs. Alginate production (µg/ml/OD_600_) was measured as described in Materials and Methods. M and NM, represent mucoidy and nonmucoidy, respectively.

CF149 is a clinical isolate from a patient with CF [24]. CF149 displays a nonmucoid phenotype on PIA and PIA plus ammonium metavanadate (PIA-AMV) plates [29]. Previously, we reported CF149 have two mutations resulting in abrogation of an AlgU-dependent transcription of lipotoxin LptF [21]. First, a frameshift mutation in *mucA* is predicted to produce a truncated MucA protein with 128 amino acids in contrast to wild type MucA with 194 amino acids. Second, CF149 carries a missense mutation in *algU* predicted to result in a substitution of alanine for valine at position 61 of the primary amino acid sequence of AlgU (AlgU^A61V^) [21]. To determine whether the alginate suppressor strain CF149 still had the ability to restore mucoidy, a transposon library was constructed and screened. As seen in Figure 1A, we identified three insertions that promote alginate production [17]. Southern blot analysis showed that only one copy of the transposon was inserted on the chromosome in these mucoid strains (data not shown). We mapped the insertion site using inverse PCR as previously described [17], [23]. Two insertions were identified in intergenic regions between PA0762 (*algU*) and PA0761 (*nadB*), and PA4428 (*sspA*) and PA4429 (probable cytochrome c1 precursor) (Figure 1A). In these two mutants, the transposon was upstream of *algU* and *sspA*, and was oriented in the same direction as the previously-observed insertion causing an overexpression of *mucE* [17]. These two mutants likely had an overexpression of *algU* and *sspA* from read through of the gentamicin-resistance gene (*aacC1*) promoter (P_Gm_) [30]. These strains were named CF149 (+*algU*) and CF149 (+*sspA*). The third mutant had an insertion site of 78 base pairs behind the stop codon of the *rpoD* gene (Figure 1A). The *rpoD* gene (PA0576) encodes the major housekeeping sigma factor (σ^70^). RpoD is essential for the growth and viability of cells, and no *rpoD* mutant has been reported in the *P. aeruginosa* mutant bank [31]. Because the orientation of the pFAC P_Gm_ is opposite to the direction of *rpoD* gene, the insertion is predicted to reduce the expression of *rpoD*. This strain was named CF149 (*−rpoD*). To verify the decreased transcription of *rpoD* in CF149 (*−rpoD*), we compared the transcript level by real-time PCR, and the activity of an RpoD-dependent promoter P*_ssrA_*-*lacZ* in two isogenic strains. The results showed that the transcript level of *rpoD* in CF149 (*−rpoD*) was 60% of the value of CF149. The Miller assay results also showed that the P*_ssrA_*-*lacZ* activity reduced by 68% in CF149 (*−rpoD*) compared to CF149. The alginate production by these mucoid strains was also measured and shown to be more than 2-fold higher than the parent strain (Figure 1B).

### SspA, not SspB, Induces Mucoidy in CF149

**Figure 2:**
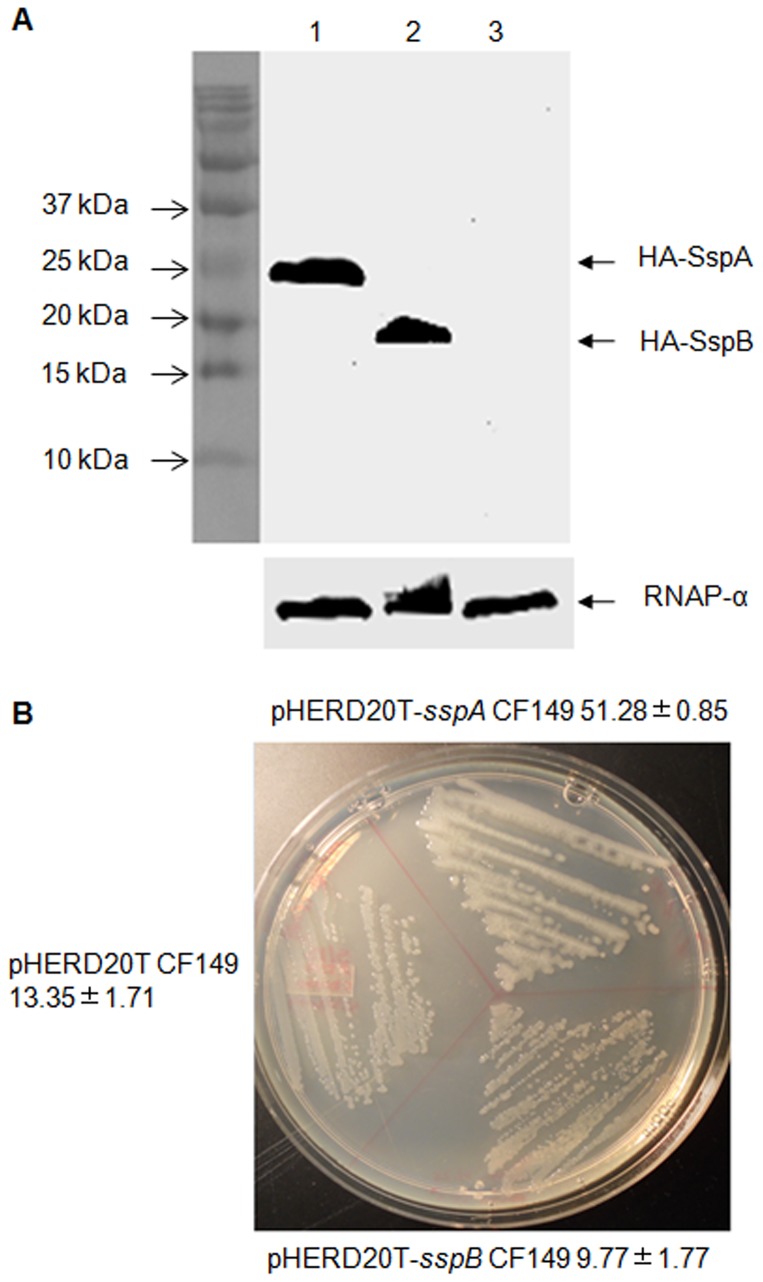
Over-expression of SspA induces mucoidy in CF149. The *sspA* and *sspB* genes from *P*. *aeruginosa* were cloned behind the P_BAD_ promoter in the pHERD20T vector, and conjugated into CF149. (A) Western blot analysis of SspA and SspB proteins using an anti-HA monoclonal antibody. Lanes 1, 2 and 3 represent total cellular proteins extracted from CF149 carrying pHERD20T-*sspA*, pHERD20T-*sspB,* and pHERD20T, respectively. (B) Morphology of CF149 containing pHERD20T-*sspA*, pHERD20T-*sspB* and pHERD20T. These strains were streaked on PIA plates supplemented with 300 µg/ml of carbenicillin, 0.1% L-Ara and incubated overnight at 37°C. Alginate production (µg/ml/OD_600_) was measured as described in Materials and Methods.

The operon of *sspAB* encodes the stringent starvation proteins A and B that function in response to amino acid starvation [32]. The *sspA* and *sspB* genes share the same promoter, and are co-expressed in *E. coli* [32]. SspB is also a specificity-enhancing factor for the protease ClpXP in *E. coli* [33]. ClpXP has been reported to regulate alginate production in *P. aeruginosa* [23]. In order to determine which gene is responsible for the mucoid conversion in CF149 (+*sspA*), *P. aeruginosa sspA* and *sspB* were cloned behind the P_BAD_ promoter in the shuttle vector pHERD20T [34]. As seen in Figure 2A, we detected the expression of SspA and SspB in CF149 after induction with 0.1% L-arabinose (L-Ara). However, we observed a mucoid phenotype only with the overexpression of *sspA* (Figure 2B), indicating that SspA is an inducer of alginate production when overexpressed in CF149.

### Anti-σ^70^ Factors Induce Conversion to Mucoidy in CF149

The mucoid phenotype expressed by CF149 (+*sspA*) and CF149 (*−rpoD*) suggests that RpoD may be involved in the alginate regulation. Schlictman *et al.* reported that *E. coli sspA* can complement the *algQ* mutation by restoring mucoidy in a CF clinical isolate [35]. AlgQ and Rsd belong to the family of the regulators of the major sigma factor RpoD (σ^70^) in Proteobacteria. Members of the anti-σ^70^ factors are thought to interact with the conserved region 4 of σ^70^ subunit of RNAP [36]. Similarly, AsiA, encoded in the T4 phage, also functions as an anti-σ^70^ factor [37]. Our hypothesis is that CF149 (+*sspA*) and CF149 (*−rpoD*) utilize the mechanism of modulating RpoD to become mucoid. To test this, we cloned the *rsd* gene from *E.coli*, *algQ* and *sspA* from *P. aeruginosa*, and *asiA* from T4 phage in pHERD20T. As seen in Table 1, overexpression of these genes caused conversion to mucoidy in CF149. However, the anti-σ^70^ factors had no effect on mucoid induction in other strains tested, even though CF4349 has a wild type *algU* and the same predicted length of MucA as CF149 (Table 1). However, mucoidy of CF149 (+*sspA*) could be due to a non-specific effect. To test this possibility, we examined the effect of anti-RpoF factor FlgM on mucoid conversion. As seen in Figure S1, FlgM had no effect on mucoid induction in PAO1 and CF149.

**Table 1:** The effect of anti-σ^70^ factors on mucoid induction in strains of *P. aeruginosa.*

Strains	MucA length	AlgU length	*rsd* (TOP 10)	*algQ* b (PAO1)	*sspA* (PAO1)	*asiA* (T4 phage)
CF149	125+3 aa a	Ala61Val (193 aa)	**M** (29.04±2.44)	**M** (28.33±2.74)	**M** (51.28±0.85)	**M** (54.51±2.28)
CF4349	125+3 aa	WT (193 aa)	NM (2.35±0.18)	NM (9.49±1.26)	NM (8.53±2.06)	NM (5.60±2.07)
CF28	117 aa	Tyr29Cys (193 aa) (193aa)	NM (6.37±2.51)	NM (8.10±2.79)	NM (13.20±0.72) (13.20±0.72) (13.20±0.72)	NM (11.56±2.05)
CF17	143+3 aa	WT (193 aa)	NM (11.45±2.97)	NM (13.52±2.64	NM (10.57±0.75)	NM (7.94±0.72)
FRD2	143+3 aa	Asp18Gly (193 aa)	NM (14.08±0.21)	NM (11.74±1.53)	NM (7.05±0.03)	NM (14.64±0.94)
PAO1	WT (194 aa)	WT (193 aa)	NM (14.48±1.56)	NM (7.84±1.67)	NM (9.38±0.3)	NM (17.76±1.46)
PA14	WT (194 aa)	WT (193 aa)	NM (9.63±1.25)	NM (8.95±1.15)	NM (8.75±0.24)	NM (14.47±2.59)
CF3715	WT (194 aa)	WT (193 aa)	NM (13.05±3.60)	NM (6.10±0.67)	NM (6.20±0.19)	NM (15.59±1.28)
CF4009	WT (194 aa)	WT (193 aa)	NM (9.21±0.49)	NM (7.24±2.31)	NM (8.69±0.11)	NM (15.64±2.52)

M and NM represent a mucoid and a non-mucoid phenotype, respectively, after incubation on PIA plates supplemented with 300 µg/ml of carbenicillin and 0.1% L-Ara at 37°C for 24 hrs. The quantity of alginate production was measured (mean ± standard error, µg/ml/OD_600_) and listed in the brackets.

a the frameshift mutation in *mucA* results in the fusion of a truncated MucA (125 amino acids of N-terminal MucA) with an additional 3 amino acids with no homology to the amino acid sequence of wild type MucA.

b a concentration of 0.5% L-Ara was needed to induce the conversion to mucoidy in CF149. The alginate production of all AlgQ-overexpressing strains was measured on PIA plates containing 300 µg/ml of carbenicillin and 0.5% L-Ara.

### Overexpression of *rpoD* Results in Suppression of Mucoidy

Mucoidy in CF149(*−rpoD*), CF149(+*sspA)* and CF149(+*algU* ) could be due to the competition between RpoD and AlgU for RNAP. To test this hypothesis, *rpoD* was cloned and overexpressed in various mucoid strains. The mucoid laboratory and clinical isolates were all suppressed by the overexpression of RpoD (Table 2). However PAO1-VE2, PAO1-VE19, PAO579 and PAO581 required a higher concentration of L-ara (0.5% vs. 0.1%) for a complete suppression of mucoidy (Table 2). To further test the hypothesis that sigma factor competition can reduce the activity of AlgU resulting in the suppression of alginate production, we induced RpoN (σ^54^), RpoS(σ^38^), and RpoF(σ^28^) in various mucoid mutants. As expected, overexpression of these sigma factors suppressed alginate production (Table S2). We also observed that overexpressed RpoD was unstable in *P. aeruginosa* and *E.coli* (Figure S2).

**Table 2:** Over-expression of RpoD suppresses mucoidy in laboratory and clinical strains of *P. aeruginosa.*

Strains		Alginate production(µg/ml/OD_600_)	MucA length	AlgU length	Alginate regulator	PHERD 20T- HA-*rpoD*-His
Laboratory strains	PAO1-VE2	**M** (64.80±6.52)	WT (194 aa)	WT (193 aa)	Over-expression of MucE	NM^a^
	PAO1-VE13	**M** (20.93±1.05)	WT (194 aa)	WT (193 aa)	Inactivation of KinB	NM
	PAO1-VE19	**M** (61.23±2.90)	WT (194 aa)	WT (193 aa)	Inactivation of MucD	NM^a^
	PAO581	**M** (58.95±3.07)	59 aa+35 aa	WT (193 aa)	MucA25	NM^a^
	PAO579	**M** (48.04±0.11)	WT (194 aa)	WT (193 aa)	PilA108	NM^a^
Clinical strains	CF149 (*−rpoD*)	**M** (30.16±0.61)	125+3 aa	Ala61Val	Reduced-expression of *rpoD*	NM
	CF149 (+*algU*)	**M** (44.20±0.49)	125+3 aa	Ala61Val	Over-expression of *algU*	NM^a^
	CF149 (+*sspA*)	**M** (25.40±1.07)	125+3 aa	Ala61Val	Over-expression of *sspA*	NM
	FRD1	**M** (56.27±4.06)	143+3 aa	WT	MucA22	NM
	PDO300	**M** (68.08±1.25)	143+3 aa	WT	MucA22	NM
	CF1003M	**M** (30.61±2.28)	59 aa+35 aa	WT (193 aa)	Unknown	NM
	CF7447M	**M** (20.22±1.40)	WT (194 aa)	WT (193 aa)	Unknown	NM

NM and M represent non-mucoidy and mucoidy, respectively, after incubation on PIA plates supplemented with 300 µg/ml of carbenicillin and 0.1% L-Ara at 37°C for 24 hrs.

a a concentration of 0.5% L-Ara was needed to completely suppress mucoidy. The values for alginate production represent an average of three independent experiments with standard error.

### Missense Mutation in CF149 *algU* Results in Production of a Variant of AlgU with Reduced Function

**Figure 3:**
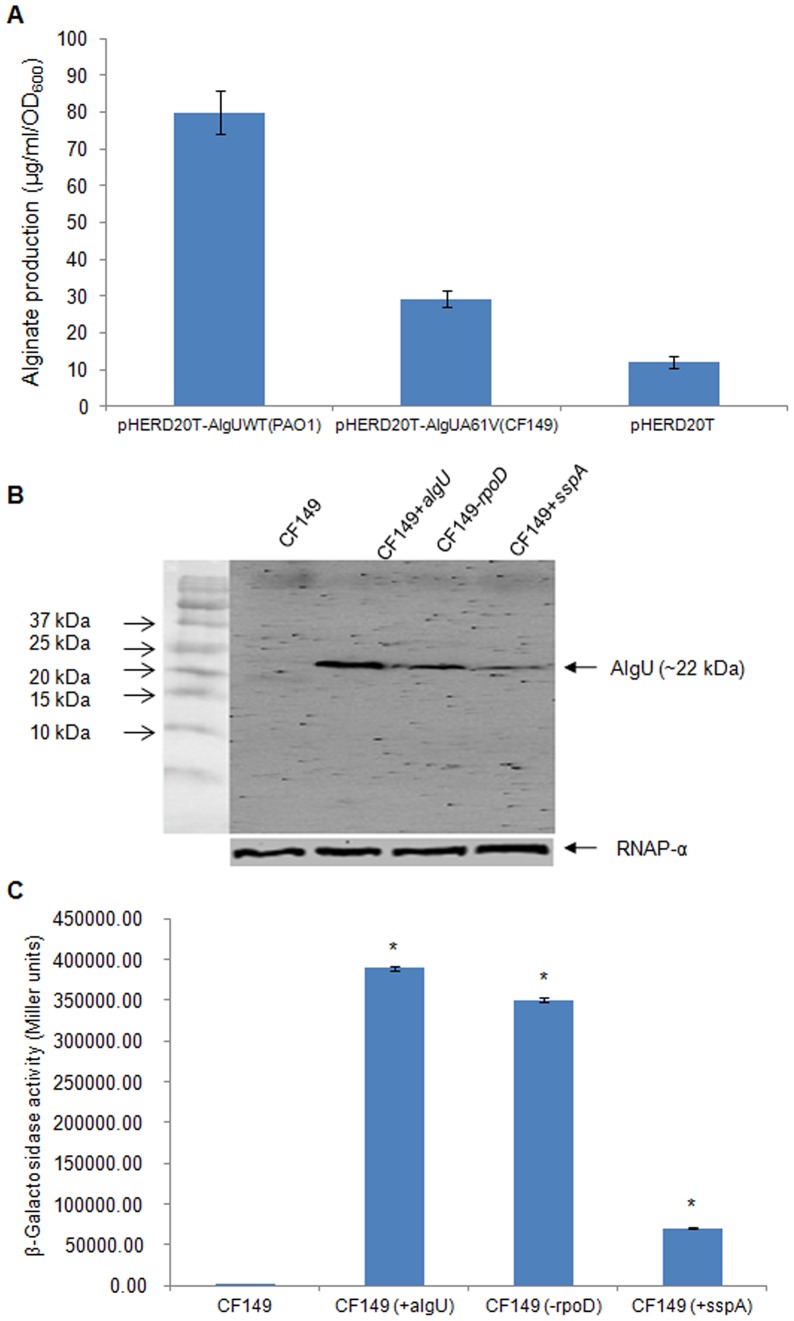
Alginate induction by CF149 AlgU^A61V^, the detection of AlgU^A61V^ and the promoter activity of P*_algD_*-*lacZ* in CF149 (+*algU*), CF149 (+*sspA*) and CF149 (*−* *rpoD*). (A) AlgU^A61V^ retained the function of inducing alginate production. The wild type *algU* gene of PAO1 and its variant of CF149 were cloned into pHERD20T, and conjugated into PAO1Δ*algU*. Strains containing pHERD20T-*algU* ^WT^ (PAO1), pHERD20T-*algU* ^A61V^ (CF149), and pHERD20T were streaked on PIA plates supplemented with 300 µg/ml of carbenicillin and incubated overnight at 37°C. Alginate production (µg/ml/OD_600_) was measured as described in Materials and Methods. (B) The level of AlgU in CF149, CF149 (+*algU*), CF149 (*−rpoD*) and CF149 (+*sspA*) was detected using Western blot with anti-AlgU monoclonal antibody [16]. (C) Measurement of the activity of the *algD* promoter in the respective strains. The P*_algD_* promoter was inserted into pLP170 vector containing the promoterless *lacZ* gene. The pLP170 P*_algD_*-*lacZ* was transferred into the respective strains via triparental conjugation. The β-galactosidase activity was measured as described in Materials and Methods. *, represents a significant difference compared to CF149 (*P*<0.05).

One explanation for mucoidy in CF149 (+*algU*) is that AlgU^A61V^ retains some function despite the amino acid substitution. To test this, we compared the function of AlgU^A61V^ vs. wild type AlgU by measuring the amount of alginate induced by two forms of AlgU in the PAO1Δ*algU* strain. CF149 AlgU^A61V^ kept the ability to induce mucoidy, albeit with a reduced amount of alginate (Figure 3A). To explain how the three mutants become mucoid, we next measured the level of AlgU^A61V^ in the total cell lysates of CF149 (+*algU*), CF149 (*−rpoD*) and CF149 (+*sspA*) through Western blot. The AlgU protein level was increased in CF149 (+*algU*), CF149 (*−rpoD*) and CF149 (+*sspA*) compared to the parent CF149 (Figure 3B). The promoter activity of P*_algD_*-*lacZ* also increased in these mucoid strains (Figure 3C).We also tested the hypothesis that the mucoidy in all three mutants was due to the increased expression of AlgU^A61V^. To do so, we introduced pHERD20T-AlgU^A61V^ into CF149. As predicted, CF149 carrying pHERD20T-AlgU^A61V^ displayed a mucoid phenotype (data not shown). Furthermore, the absence of AlgU^A61V^ in Figure 3B is consistent with non-mucoidy of CF149 on PIA, which is probably due to the reduced auto-regulatory activity of AlgU^A61V^ [38].

### Intramembrane Proteolysis has a Minimal Role in the Regulation of Mucoidy in CF149

**Figure 4:**
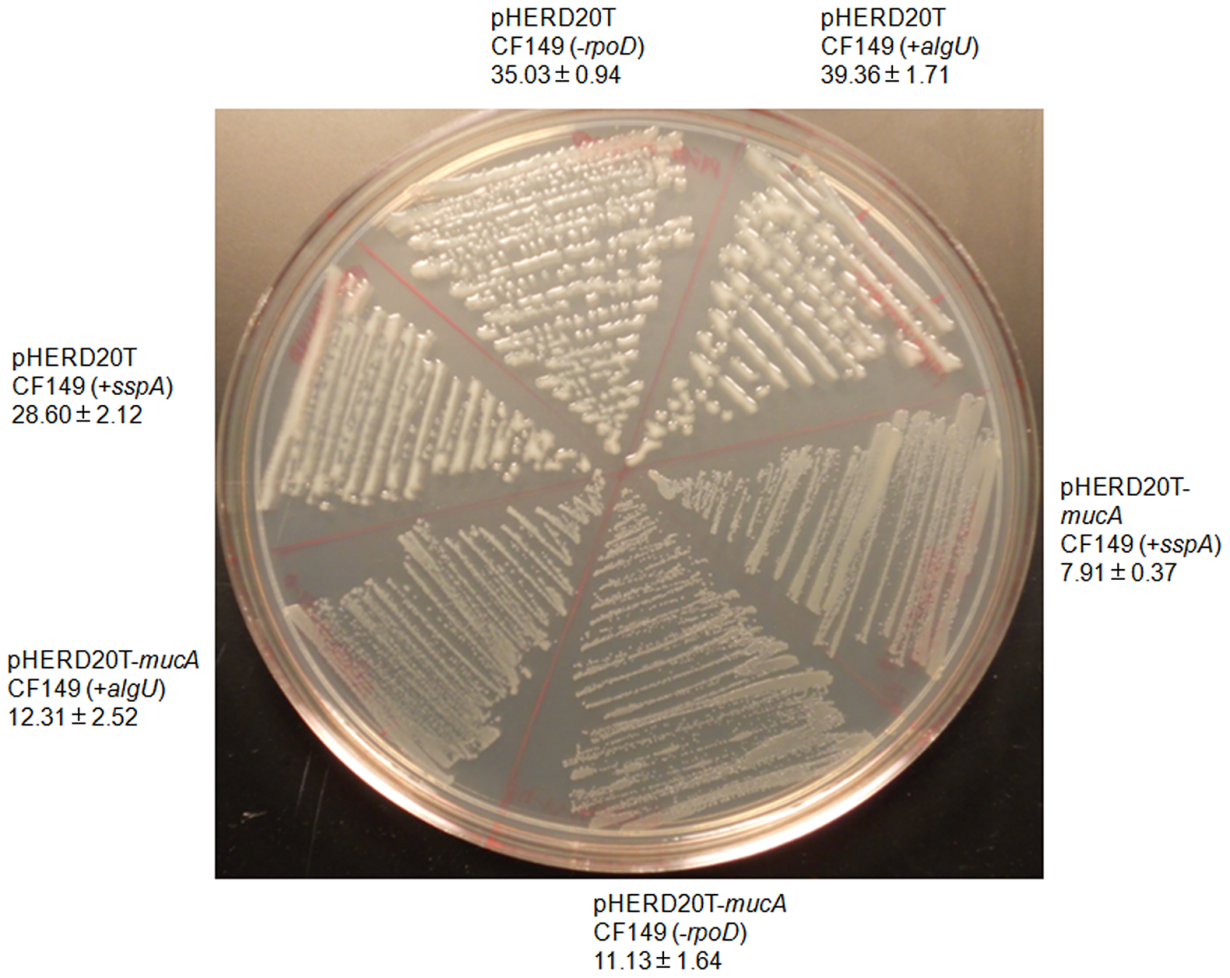
The effect of *mucA* on the mucoid suppression in CF149 (+*algU*), CF149 (+*sspA*) and CF149 (*−* *rpoD*). AlgU^A61V^–induced mucoidy was suppressed by the wild type *mucA in trans*. The *mucA* gene was over-expressed *in trans* in CF149(+*algU*), CF149(*−rpoD*) and CF149(+*sspA*) by adding 0.1% L-Ara in PIA supplemented with 300 µg/ml carbenicllin. The plate was incubated for 24 hrs at 37°C.

In wild type strain PAO1, AlgU can be sequestered by wild type MucA, thereby preventing it from activating the alginate biosynthetic operon [12], [38], [39]. Since all three mutants of CF149 have an increased level of AlgU^A61V^, we next investigated whether the wild type MucA can still exert an inhibitory effect on AlgU^A61V^. The wild type *mucA* gene was transferred into these mucoid strains: CF149 (+*algU*), CF149 (+*sspA*) and CF149 (*−rpoD*). Over-expression of *mucA* suppressed mucoidy (Figure 4) suggesting that the mucoidy of all three mutants is due to the activation of the AlgU pathway. But the mutant MucA in CF149 has 128 amino acid residues, and carries the intact trans-membrane domain of MucA_84–104_. We also noticed that the promoter activity of P*_algW_* in CF149 (*−rpoD*) was increased compared to CF149 (Figure S3). To test if intramembrane proteolysis has a role in cleaving the periplasmic portion of MucA in CF149, we overexpressed proteases *algW*, *mucP*, *clpX*, *clpP* and *clpP2* and found this had no effect on mucoid induction in CF149. Together, these results suggest that these proteases have a minimal role in regulating the mucoid conversion in CF149, or they may require a mechanism of activation which is absent in CF149.

### Competition between RpoD- and AlgU-dependent Promoters in Mucoid and Non-mucoid Strains

**Figure 5:**
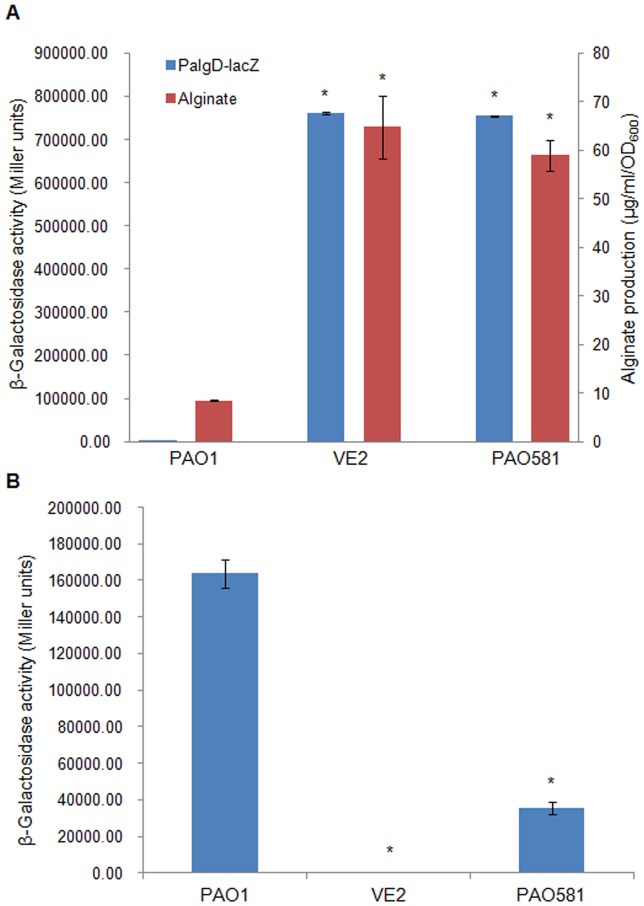
Regulation of RpoD- and AlgU-dependent promoters in isogenic non-mucoid and mucoid strains of *P. aeruginosa*. (A) The β-galactosidase activity of AlgU-dependent promoter P*_algD_*-*lacZ* and alginate production (µg/ml/OD_600_) were measured in non-mucoid and mucoid strains. (B) The β-galactosidase activity of an RpoD-dependent promoter P*_ssrA_*-*lacZ* was measured in non-mucoid and mucoid strains. *, represents a significant difference compared to PAO1 (*P*<0.05).

**Figure 6:**
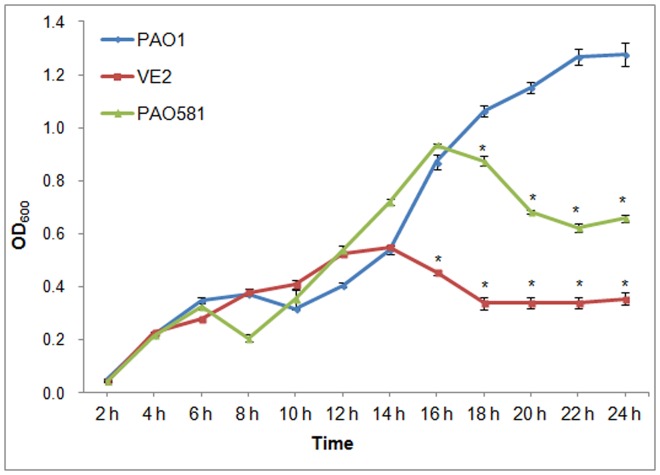
Mucoid mutants of *P. aeruginosa* display a reduced growth rate compared to the isogenic nonmucoid strain PAO1. The growth curves were created by growth of PAO1 (nonmucoid), VE2 (mucoid) and PAO581 (mucoid) in PIB. The horizontal axis represents time in hrs, while the vertical axis is the optical density at 600 nm. *, represents a significant difference compared to PAO1 (*P*<0.05).

Since the transcription from two promoters, P*_algD_* and P*_ssrA_* is AlgU-dependent and RpoD-dependent, respectively, we cloned these two promoters into a *lacZ* fusion vector (pLP170) and the reporter β-galactosidase activity was measured [26]. As seen in Figure 5A, alginate production in the mucoid strains VE2 and PAO581 was higher than that in the non-mucoid strain PAO1. Similarly, the promoter activity of P*_algD_* also was higher in these mucoid strains (Figure 5A). However, the promoter activity of P*_ssrA_* was inversely related to P*_algD_* activity and alginate production (Figure 5B), suggesting that the RpoD-dependent promoter was less active in mucoid cells than in the isogenic non-mucoid cells. We also measured the growth curve for mucoid and non mucoid strains. As seen in Figure 6, the growth rate for mucoid strains was reduced after 16 hrs growth for VE2 and PAO581 in comparison with nonmoucid strain PAO1.

## Discussion

**Figure 7:**
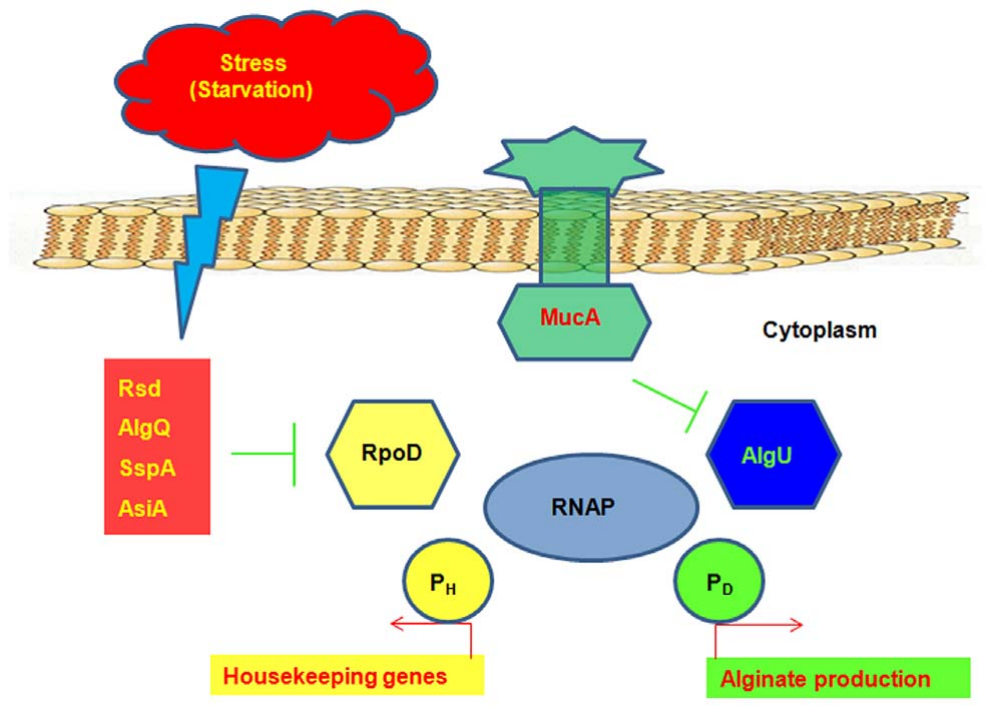
Schematic diagram for mucoid conversion in CF149 caused by competition between two sigma factors, RpoD and AlgU. Stress or starvation can affect the expression of anti-σ^70^ factors resulting in a decrease in the activity of RpoD, which in turn increases the chances for the alternative sigma factor AlgU to bind to the core RNAP to initiate transcription for alginate biosynthesis. MucA is an anti-sigma factor that has the ability to inhibit the activity of AlgU in alginate production. Competition for RNAP between RpoD and AlgU can determine which promoter is activated. RpoD binds to core RNAP to promote the transcription of housekeeping genes, while AlgU binds to core RNAP at the P*_algD_* promoter to activate alginate production.

Individuals with CF are thought to acquire initial colonization of *P. aeruginosa* from environmental sources [9]. These early colonizing strains display a non-mucoid phenotype with a wild-type MucA [17]. Due to strong selective pressure in CF lungs, mucoid *mucA* mutants eventually become a dominant population [14], [40]. However, secondary mutations that suppress alginate overproduction have been reported [8], [19]. One presumed advantage with non-mucoid suppressors is the loss of mucoid status is coupled with the presence of the flagella, which may promote the colonization of new niches in the lungs [41], [42]. Through screening a transposon library, we found that overexpression of *sspA* and CF149 *algU*, and reduced expression of *rpoD*, are functionally equivalent in causing mucoid conversion in the non-mucoid clinical isolate CF149. This mucoid phenotype can be suppressed by overexpression of the anti-sigma factor MucA [12]. We propose that the mechanism for mucoid conversion mediated by AlgU, SspA and RpoD in CF149 may be related to the competition between sigma factors RpoD and AlgU for the core RNAP binding site (Figure 7). Because of the differential binding ability among sigma factors for core RNAP [43], σ factor competition exists within a cell at any given time [44]. We investigated whether this competition is also present in CF149 and CF149(*−rpoD*), by measuring the promoter activity of P*_algw_* whose activation depends on RpoN [45], and P*_algD_* which is driven by AlgU [11]. As seen in Figures 3C and S3, activities of both P*_algD_* and P*_algW_* were increased in CF149 (*−rpoD*). Furthermore, the mucoid suppression resulting from the overexpression of RpoD can be attributed to the competition between two sigma factors (Table 2). Table S2 illustrates that sigma factors besides RpoD can also exert the same effect on mucoid suppression in *mucA* plus and minus mucoid strains. Thus, any major shift in the intracellular level of sigma factors can potentially affect mucoid conversion, because the pool of core RNAP, which is made up of five different subunits, must be in a limiting amount inside bacterial cells. However, data in Figure S1 demonstrate that induction of FlgM, which is the anti-sigma factor for RpoF responsible for the transcription initiation of flagella biosynthesis [46], failed to induce mucoidy in PAO1 and CF149. This may be due to the fact that the impact on the pool of RNAP is somewhat different between a minor sigma factor RpoF and a major sigma factor RpoD. Therefore not all anti-sigma factors are functionally equal in terms of alginate induction.

Anti-sigma factors are proteins that bind to cognate sigma factors, thereby inhibiting their transcriptional activity [47]. The *E.coli* protein Rsd associates specifically with σ^70^ to inhibit the σ^70^-dependent transcription [48]. The *P. aeruginosa* transcriptional regulator AlgQ/AlgR2, shares 55% identity at the amino acid sequence level with Rsd [48], and has been proposed as an anti-σ^70^ factor that interacts with σ^70^ to affect the transcriptional activity of *algD* [49]. The T4 phage anti-σ^70^ factor AsiA also has a similar function as Rsd and AlgQ. Specifically, AsiA can regulate the transcriptional activity of *rpoD* and other promoters [37], [50], [51], [52]. Although the amino acid sequence similarity is low (<10%) between *P. aeruginosa* SspA to anti-σ^70^ factors Rsd, AsiA and AlgQ, the similarity at amino acid level between *E. coli* SspA to *P. aeruginosa* SspA is higher than 50%. Hansen *et al* reported that *E. coli* SspA is an RNAP-associated protein, and can down-regulate expression from the σ^70^-dependent promoters [53]. More importantly, *E. coli* SspA can functionally replace *P. aeruginosa* anti-sigma factor AlgQ [35]. The sequence diversity in anti-sigma factors suggests that they may have different binding sites on RpoD [36]. Their inhibitory activity to the σ^70^-driven transcription is therefore different. Compared with the strong inhibition of AsiA, Rsd shows only a modest effect on σ^70^ transcription *in vitro* and *in vivo* [48], [54], [55]. This may explain why overexpression of Rsd and AsiA can induce mucoidy in CF149 when the growth media are supplemented with 0.1% L-Ara, while AlgQ-induced mucoidy in this strain requires a higher concentration of 0.5% L-Ara (Table 1). Also, compared with Rsd and AsiA, AlgQ does not have the ability to significantly inhibit the growth of *E. coli in vivo* [54].

In the current study, anti-σ^70^ factors were found to have an effect on mucoid induction in suppressed nonmucoid strain CF149. This induction is not directly through the interaction with the core RNAP, rather it is through the reduction of RpoD activity. CF149 may be a rare alginate suppressor mutant. The amino acid substitution in AlgU^A61V^ in CF149 was mapped to the conserved region of Sigma 70_r2 Superfamily [NCBI CDD cl08419]. This region contains both the −10 promoter recognition helix and the primary core RNAP binding determinant. However, the change from alanine to valine is of conservative nature (non-polar to non-polar), which may explain why this mutation renders AlgU^A61V^ partially defective. Therefore, it is not surprising to see that not all clinical isolates respond to the induction by anti-σ^70^ factors in the same manner (Table 1). For example, CF4349 is not inducible, even though it has a wild type AlgU and the *mucA* genotype is the same as CF149. This suggests that there could be another unknown suppressor mutation, which nullifies the effect of anti-σ^70^ induction in CF4349, or the mutation in CF149 AlgU^A61V^ somehow amplifies the effect of anti-σ^70^ induction.

Earlier work showed that the growth condition that promotes the fast growth rate of *P. aeruginosa* PAO1 does not select for the mucoid variants, but the condition that caused a slow growth rate does [56]. In *Salmonella*, the expression of RpoE, the AlgU homolog induced the stationary phase of growth (slow growth rate) [57]. In the current study, overexpression of anti-RpoD factors can induce mucoidy in CF149 and overexrpression of RpoD can inhibit mucoidy in all tested strains. Furthermore, there is a competition between RpoD- and AlgU-dependent promoters (Figure 5), and the growth rate of both mucoid strain VE2 and PAO581 were slower than that of the isogenic nonmucoid strain PAO1 (Figure 6). These data provide genetic evidence for the sigma factor competiton in the regulation of alginate production by *P. aeruginosa*.

Mucoid conversion in *P. aeruginosa* can be achieved through two known mechanisms. Intra-membrane proteolysis acts as a primary mechanism to initiate the degradation of MucA in *P. aeruginosa* with wild type *mucA* [13]. Mutation of the *mucA* gene is another mechanism to become mucoid and is prevalent in isolates from chronic lung infections in CF [2]. In the current study, we showed that a missense mutation in *algU* causes the loss of mucoidy in the *mucA* mutant, but augmentation of anti-σ^70^ factors such as SspA leads to increased amounts of AlgU, causing mucoid conversion in the AlgU-suppressed strain. Williams *et al* reported that carbon, amino acid, nitrogen and phosphate starvation can induce the expression of SspA [32], and Roychoudhury *et al* found that starvation caused by nitrogen and phosphate limitation is one of the signals leading to AlgQ-mediated activation of the *algD* promoter in *P. aeruginosa* [58]. Therefore, stress signals may activate the expression of anti-RpoD factors, thus causing mucoid conversion in the suppressed strains.

In summary, we found three genes *algU*, *sspA* and *rpoD* that regulate the conversion to mucoidy in the MucA-truncated, non-mucoid *P. aeruginosa* strain CF149, which contains an AlgU-suppressor mutation. Interestingly, our data indicate the missense mutation in CF149 AlgU only reduces, but does not completely abolish the function as the sigma factor that drives alginate biosynthetic operon. The mechanism by which these genes cause mucoidy may be due to competition between the sigma factors AlgU and RpoD. Also, anti-σ^70^ factors AsiA, Rsd, AlgQ and SspA can induce mucoidy in strain CF149.

## Acknowledgments

We thank Richard M. Niles for assistance in revising this manuscript, and Gary Schultz from the Department of Biology at Marshall University for providing the T4 bacteriophage and its host *E. coli* BB.

## Supporting Information

Figure S1 **FlgM, an anti-sigma factor for RpoF, fails to induce mucoidy in** **CF149 and PAO1.** pHERD20T-*flgM* was conjugated into CF149 and PAO1, respectively. Strains carrying pHERD20T-*flgM,* pHERD20T-*sspA*, and pHERD20T were incubated on PIA plates supplemented with 300 µg/ml carbenicillin, 0.1% L-ara and incubated at 37°C for 24 hrs. Alginate was harvested and measured as described in Materials and Methods. *, represents a significant difference between each group (*P*<0.05).
(TIF)

Figure S2 **The stability of over-expressed RpoD in** ***P. aeruginosa*** **and** ***E.coli.*** RpoD was expressed in *P. aeruginosa* PAO581 (A) and *E. coli* TOP10 cells (B) carrying pHERD20T-HA-*rpoD*-His under the induction with different concentration of L-Ara. PAO581 carried pHERD20T-HA-*rpoD*-His was cultured on PIA plates supplemented with 300 µg/ml carbenicllin and different concentrations of L-Ara for 24 hrs and the cells were then collected for cell lysis. TOP10 carried pHERD20T-HA-*rpoD*-His was cultured on LB plates supplemented with 100 µg/ml carbenicllin and L-Ara for 24 hours. Following sonication, 50 µg protein of total cell lysate from each sample was used for SDS-PAGE and Western blotting analysis. Lane 1: the protein molecular mass standards; Lane 2: cells no L-Ara; Lane 3: cells induced by adding 0.1% L-Ara; Lane 4: cells induced by adding 0.5% L-Ara; Lane 5: control with the empty vector.
(TIF)

Figure S3 **Reduced expression of RpoD in CF149(** ***−rpoD*** **) is correlated with increased promoter activity of P** ***_algW_*** **.** RpoN dependent promoter pLP170-P*_algW_* was conjugated into CF149 and CF149 (*−rpoD*), respectively. The Miller assay was used to detect the activation of P*_algW_* in these strains. *, represents the difference of the β-galactosidase activity between these strains is significant (*P*<0.05).
(TIF)

Table S1 **Strains and plasmids used in this study.**
(DOC)

Table S2 **Inhibitory effect of sigma factors RpoN, RpoS and RpoF on** ***P. aeruginosa*** **mucoidy.**
(DOC)
