# Supplementary figures and images for: Evidence for Sigma Factor Competition in the Regulation of Alginate Production by Pseudomonas aeruginosa
Source: PLoS One. 2013 Aug 22;8(8):e72329. doi: 10.1371/journal.pone.0072329 (PMC3750012; doi:10.1371/journal.pone.0072329)

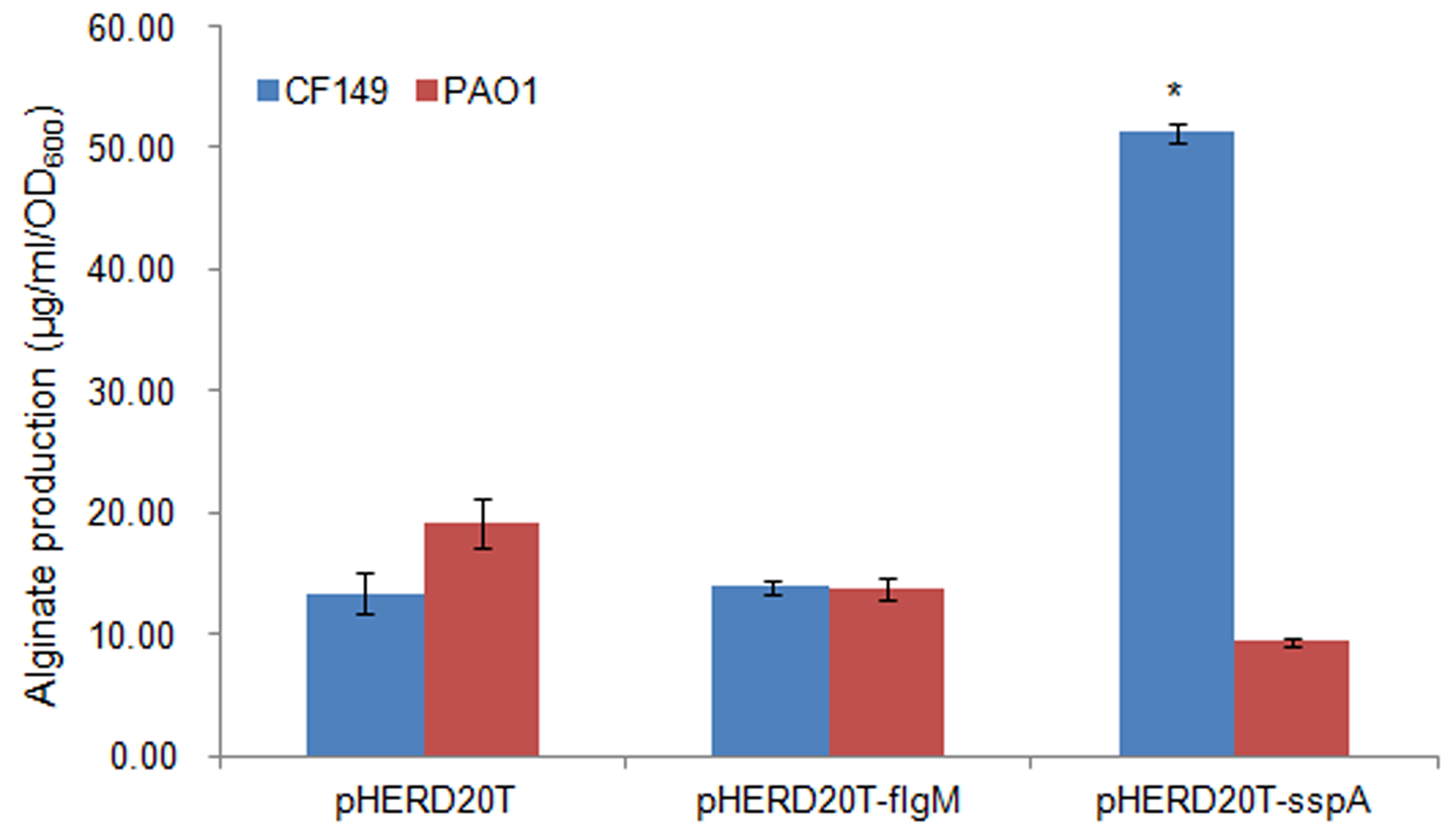

Supplement: Figure S1 — FlgM, an anti-sigma factor for RpoF, fails to induce mucoidy in CF149 and PAO1. pHERD20T-flgM was conjugated into CF149 and PAO1, respectively. Strains carrying pHERD20T-flgM, pHERD20T-sspA, and pHERD20T were incubated on PIA plates supplemented with 300 µg/ml carbenicillin, 0.1% L-ara and incubated at 37°C for 24 hrs. Alginate was harvested and measured as described in Materials and Methods. *, represents a significant difference between each group (P<0.05). (TIF) [file pone.0072329.s001.tif]

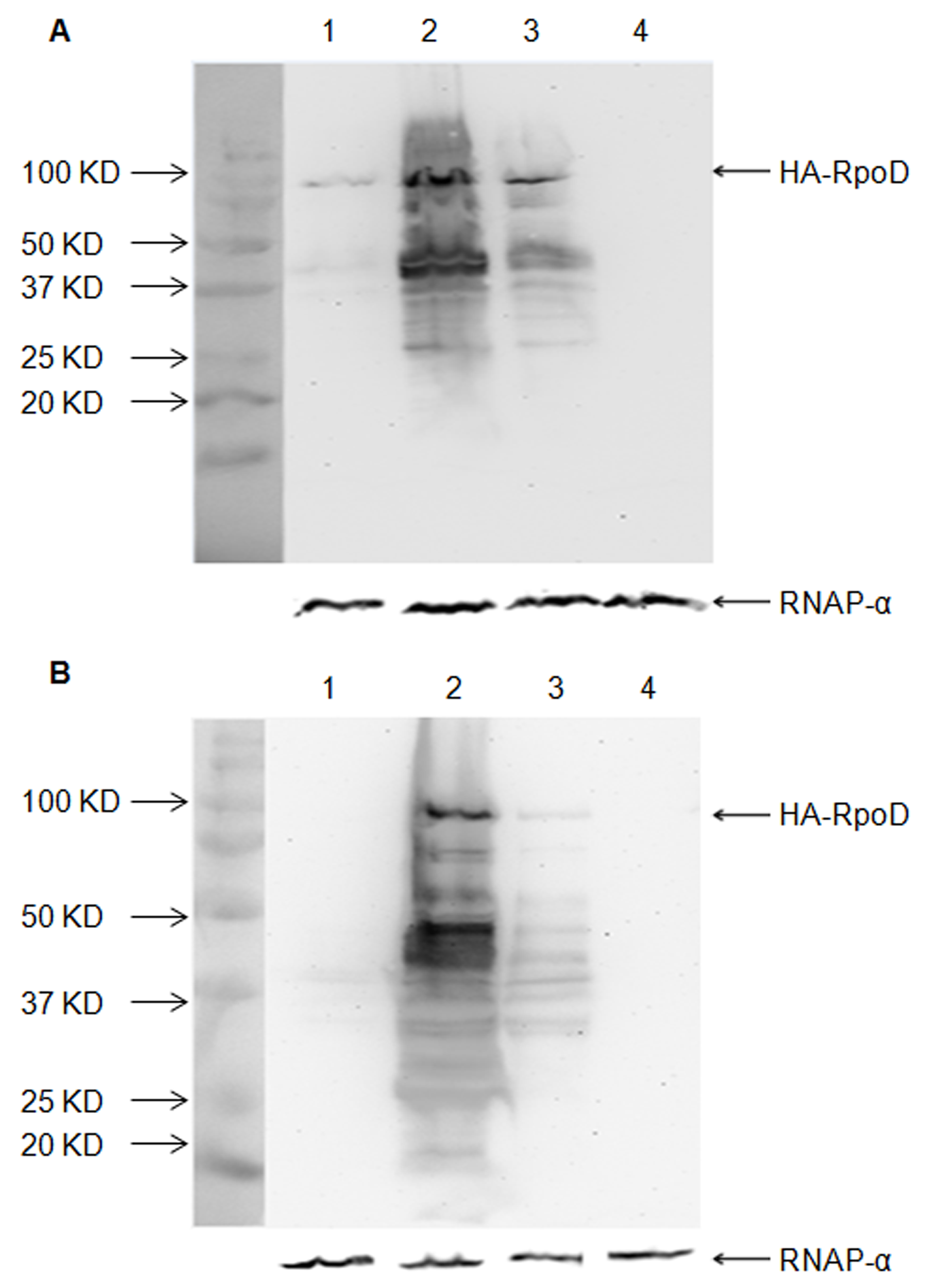

Supplement: Figure S2 — The stability of over-expressed RpoD in P. aeruginosa and E.coli. RpoD was expressed in P. aeruginosa PAO581 (A) and E. coli TOP10 cells (B) carrying pHERD20T-HA-rpoD-His under the induction with different concentration of L-Ara. PAO581 carried pHERD20T-HA-rpoD-His was cultured on PIA plates supplemented with 300 µg/ml carbenicllin and different concentrations of L-Ara for 24 hrs and the cells were then collected for cell lysis. TOP10 carried pHERD20T-HA-rpoD-His was cultured on LB plates supplemented with 100 µg/ml carbenicllin and L-Ara for 24 hours. Following sonication, 50 µg protein of total cell lysate from each sample was used for SDS-PAGE and Western blotting analysis. Lane 1: the protein molecular mass standards; Lane 2: cells no L-Ara; Lane 3: cells induced by adding 0.1% L-Ara; Lane 4: cells induced by adding 0.5% L-Ara; Lane 5: control with the empty vector. (TIF) [file pone.0072329.s002.tif]

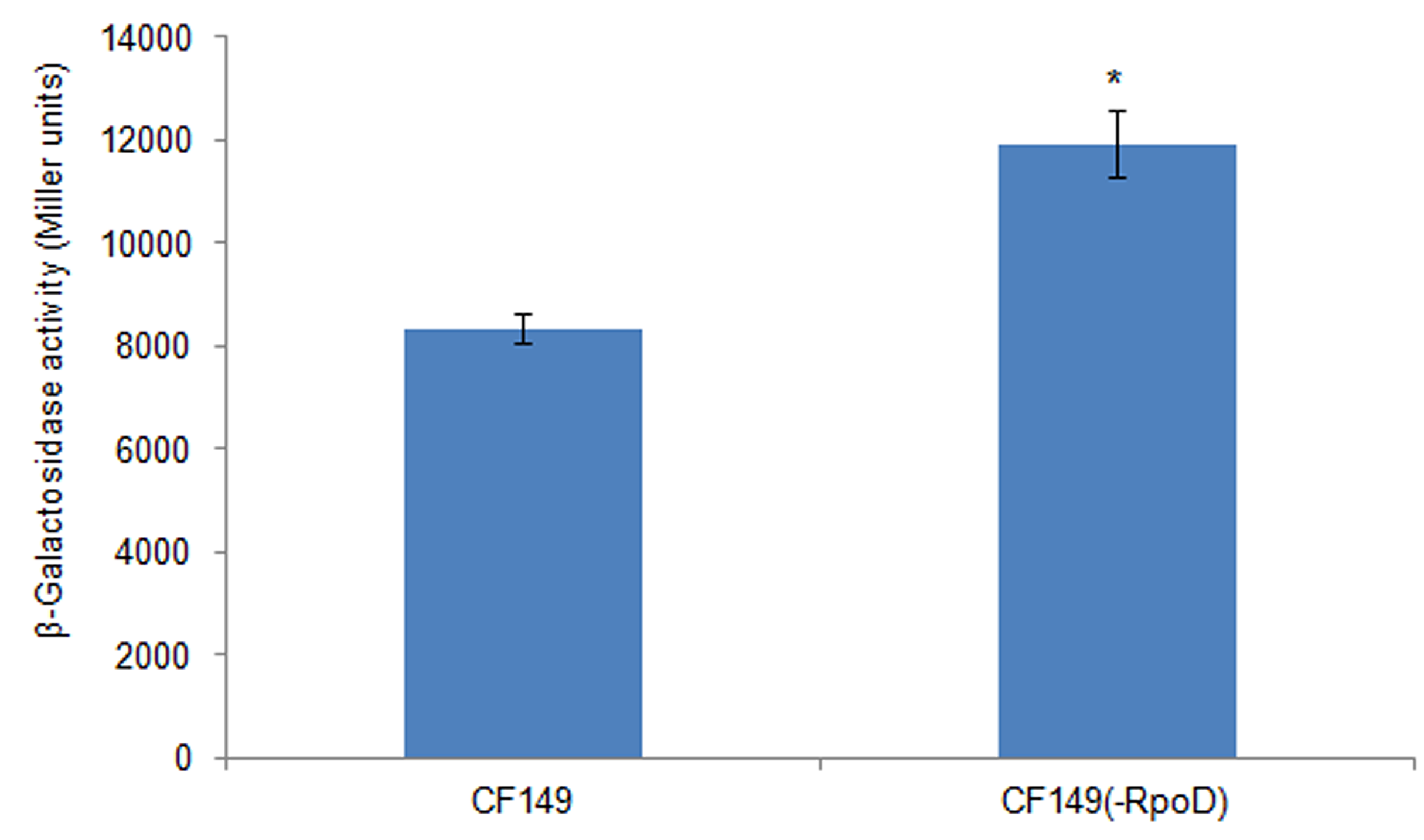

Supplement: Figure S3 — Reduced expression of RpoD in CF149( −rpoD ) is correlated with increased promoter activity of P algW . RpoN dependent promoter pLP170-PalgW was conjugated into CF149 and CF149 (−rpoD), respectively. The Miller assay was used to detect the activation of PalgW in these strains. *, represents the difference of the β-galactosidase activity between these strains is significant (P<0.05). (TIF) [file pone.0072329.s003.tif]
