# Supplementary material for: Evidence for Sigma Factor Competition in the Regulation of Alginate Production by Pseudomonas aeruginosa
Source: PLoS One. 2013 Aug 22;8(8):e72329. doi: 10.1371/journal.pone.0072329 (PMC3750012; doi:10.1371/journal.pone.0072329)
Supplement: Table S1 — Strains and plasmids used in this study. (DOC) [file pone.0072329.s004.doc]

**Table S1. Strains and plasmids used in this study.**

| Strain and plasmid | Phenotype and genotype | Source or reference |
| --- | --- | --- |
| ***P. aeruginosa* strains** |  |  |
| CF149 | Non-mucoid, *mucA* mutant (128 aa), *algU* suppressor mutant (A61V) | G. Pier^a^ |
| CF149 (-*rpoD*) | Mucoid, reduced expression of *rpoD*, Gm^R^, CF149 | This study |
| CF149 (+*algU*) | Mucoid, overexpression of *algU*, Gm^R^, CF149 | This study |
| CF149 (+*sspA*) | Mucoid, overexpression of *sspA*, Gm^R^, CF149 | This study |
| PAO1 | Non-mucoid, prototroph | P. Phibbs^b^ |
| PA14 | Non-mucoid, prototroph | F. Ausubel^a^ |
| PDO300 | Mucoid, *algT^+^mucA22* in PAO1 |  |
| FRD1 | Mucoid, *algT^+^mucA22,* clinical strain |  |
| FRD2 | Non-mucoid, *algT* suppressor mutation, FRD1 |  |
| CF17 | Non-mucoid, *mucA* mutant, *algU* wild type, derived from clinical mucoid strain | This study |
| CF28 | Non-mucoid, *mucA* mutant, *algU* mutant, derived from clinical mucoid strain | This study |
| CF4349 | Non-mucoid, *mucA* mutant, *algU* wild type, derived from clinical mucoid strain | This study |
| CF3715 | Non-mucoid, *algU^+^mucA^+^*, isolated from early lung colonization in CF |  |
| CF4009 | Non-mucoid, *algU^+^mucA^+^*, isolated from early lung colonization in CF | D. Speert^c^ |
| CF1003 | Mucoid, *algU^+^ mucA^-^*, isolated from the lung of a CF mouse |  |
| CF7447 | Mucoid, *algU^+^ mucA^+^*, isolated from early lung colonization in CF. | D. Speert^c^ |
| PAO1-VE2 | Mucoid, *algU^+^ mucA^+^*,*mucE*-overexpressing, Gm^R^, PAO1 |  |
| PAO1-VE13 | Mucoid, *algU^+^ mucA^+^*, *kinB* mutant, Gm^R^, PAO1 |  |
| PAO1-VE19 | Mucoid, *algU^+^ mucA^+^*, *mucD* mutant, Gm^R^, PAO1 |  |
| PAO579 | Mucoid, *algU^+^mucA^+^*, derived from PAO381, and PAO1 |  |
| PAO581 | Mucoid, PAO1 *algU^+^ mucA25* |  |
| PAO581Δ*algU* | Non-mucoid, PAO581Δ*algU*, in-frame deletion of *algU* |  |
| PAO1Δ*algU* | PAO1Δ*algU,* an in-frame deletion of *algU* |  |
| ***E. coli* strains** |  |  |
| TOP10 | DH5α derivative | Invitrogen |
| SM10/λpir | *thi thr leu tonA lacY supE recA :: RP4-2-Tc :: Mu lpir* Km^R^ | Laboratory strain |
| *E. coli* BB | Host cell for T4 phage | G. Shultz^e^ |
| **Plasmids** |  |  |
| pFAC | Mini-*himar*I mariner transposon with a selectable marker Gm^R^ Ap^R^ |  |
| pRK2013 | Km^R^ Tra Mob ColE1 |  |
| pHERD 20T | pUCP20T P_lac_ replaced by fragment of *araC*–P_BAD_ cassette |  |
| pLP170 | Promoterless *lacZ* fusion vector, Ap^R^ | L. Passador^f^ |
| pHERD 20T-HA-*rpoD*-His | *rpoD* (PA0576) from PAO1 in pHERD20T EcoRI/HindIII | This study |
| pHERD 20T-HA-*sspA*-His | *sspA* (PA4428) from PAO1 in pHERD20T NcoI/EcoRI | This study |
| pHERD 20T-HA-*sspB*-His | *sspB* (PA4427) from PAO1 in pHERD20T EcoRI/HindIII | This study |
| pHERD 20T-*algQ*-His | *algQ* (PA5255) from PAO1 in pHERD20T EcoRI/HindIII | This study |
| pHERD 20T-HA-*rsd*-His | *rsd* from TOP10 in pHERD20T NcoI/EcoRI | This study |
| pHERD 20T-HA-*asiA*-His | *asiA* from E. coli T4 phage in pHERD20T NcoI/EcoRI | This study |
| pHERD 20T-HA-*mucA*-FLAG | *mucA* (PA0763) from PAO1 in pHERD20T EcoRI/HindIII | This study |
| pHERD 20T-*algW*-His | *algW* (PA4446) from PAO1 in pHERD20T EcoRI/HindIII | This study |
| pHERD 20T-HA-*mucP*-His | *mucP* (PA3649) from PAO1 in pHERD20T NcoI/HindIII | This study |
| pHERD 20T-HA-*clpX*-His | *clpX* (PA1802) from PAO1 in pHERD20T NcoI/HindIII | This study |
| pHERD 20T-*clpP*-His | *clpP* (PA1801) from PAO1 in pHERD20T EcoRI/HindIII | This study |
| pHERD 20T-*clpP2*-His | *clpP2* (PA3326) from PAO1 in pHERD20T EcoRI/HindIII | This study |
| pHERD 20T-*algU*^WT^(PAO1) | *algU* (PA0762) from PAO1 in pHERD20T EcoRI/HindIII |  |
| pHERD 20T-*algU*^A61V^(CF149) | *algU* (PA0762) from CF149 in pHERD20T EcoRI/HindIII | This study |
| pLP170-P*_algD_* | Promoter of *algD* (PA3540) from PAO1 in pLP170 EcoRI/HindIII |  |
| pLP170-P*_ssrA_* | Promoter of *ssrA* from *E. coli* TOP10 in pLP170 EcoRI/HindIII | This study |
| pLP170-P*_algW_* | Promoter of *algW* (PA4446) from PAO1 in pLP170 EcoRI/HindIII |  |

^a^, Harvard Medical School, USA; ^b^, East Carolina University, USA; ^c^, University of British Columbia, CA; ^d^, University of Edinburgh, UK; ^e^, Marshall University, USA; ^f^, University of Rochester, USA.

**References:**

1. Mathee K, Ciofu O, Sternberg C, Lindum PW, Campbell JI, et al. (1999) Mucoid conversion of *Pseudomonas aeruginosa* by hydrogen peroxide: a mechanism for virulence activation in the cystic fibrosis lung. Microbiology 145 ( Pt 6): 1349-1357.

2. Ohman DE, Chakrabarty AM (1981) Genetic mapping of chromosomal determinants for the production of the exopolysaccharide alginate in a *Pseudomonas aeruginosa* cystic fibrosis isolate. Infect Immun 33: 142-148.

3. Olson JC, Ohman DE (1992) Efficient production and processing of elastase and LasA by *Pseudomonas aeruginosa* require zinc and calcium ions. J Bacteriol 174: 4140-4147.

4. Qiu D, Eisinger VM, Rowen DW, Yu HD (2007) Regulated proteolysis controls mucoid conversion in *Pseudomonas aeruginosa*. Proc Natl Acad Sci U S A 104: 8107-8112.

5. Qiu D, Eisinger VM, Head NE, Pier GB, Yu HD (2008) ClpXP proteases positively regulate alginate overexpression and mucoid conversion in *Pseudomonas aeruginosa*. Microbiology 154: 2119-2130.

6. Damron FH, Qiu D, Yu HD (2009) The *Pseudomonas aeruginosa* sensor kinase KinB negatively controls alginate production through AlgW-dependent MucA proteolysis. J Bacteriol 191: 2285-2295.

7. Damron FH, Yu HD (2011) *Pseudomonas aeruginosa* MucD regulates the alginate pathway through activation of MucA degradation via MucP proteolytic activity. J Bacteriol 193: 286-291.

8. Govan JR, Fyfe JA (1978) Mucoid *Pseudomonas aeruginosa* and cystic fibrosis: resistance of the mucoid from to carbenicillin, flucloxacillin and tobramycin and the isolation of mucoid variants in vitro. J Antimicrob Chemother 4: 233-240.

9. Damron FH, Davis MR, Jr., Withers TR, Ernst RK, Goldberg JB, et al. (2011) Vanadate and triclosan synergistically induce alginate production by *Pseudomonas aeruginosa* strain PAO1. Mol Microbiol 81: 554-570.

10. Figurski DH, Helinski DR (1979) Replication of an origin-containing derivative of plasmid RK2 dependent on a plasmid function provided in trans. Proc Natl Acad Sci U S A 76: 1648-1652.

11. Qiu D, Damron FH, Mima T, Schweizer HP, Yu HD (2008) PBAD-based shuttle vectors for functional analysis of toxic and highly regulated genes in *Pseudomonas* and *Burkholderia spp.* and other bacteria. Appl Environ Microbiol 74: 7422-7426.

12. Ryan Withers T, Heath Damron F, Yin Y, Yu HD (2013) Truncation of type IV pilin induces mucoidy in *Pseudomonas aeruginosa* strain PAO579. Microbiologyopen 2: 459-470.
