# Supplementary material for: Evidence for Sigma Factor Competition in the Regulation of Alginate Production by Pseudomonas aeruginosa
Source: PLoS One. 2013 Aug 22;8(8):e72329. doi: 10.1371/journal.pone.0072329 (PMC3750012; doi:10.1371/journal.pone.0072329)
Supplement: Table S2 — Inhibitory effect of sigma factors RpoN, RpoS and RpoF on P. aeruginosa mucoidy. (DOC) [file pone.0072329.s005.doc]

**Table S2. Inhibitory effect of sigma factors RpoN, RpoS and RpoF on *P. aeruginosa* mucoidy.**

| Strains | Mucoid conversion factor | Alginate production *  µg/ml/OD_600_ | pHERD20T-*rpoN* | | pHERD20T-*rpoS* | | pHERD20T-*rpoF* | |
| --- | --- | --- | --- | --- | --- | --- | --- | --- |
|  |  |  | 0.1% L-ara | 0.5% L-ara | 0.1% L-ara | 0.5% L-ara | 0.1% L-ara | 0.5% L-ara |
| PAO1-VE2 | Over-expression of MucE | 64.80±11.29 | M | NM | M | NM | NM | NM |
| PAO581 | MucA25 | 58.95±5.32 | NM | NM | M | NM | NM | NM |
| FRD1 | MucA22 | 56.27±4.06 | NM | NM | M | NM | NM | NM |
| PDO300 | MucA22 | 68.08±1.25 | M | M | M | NM | NM | NM |
| CF149(+*algU*) | Over-expression of *algU* | 44.20±0.49 | M | M | M | NM | M | NM |

NM and M represent non-mucoidy and mucoidy, respectively.

*, the alginate production was measured before pHERD 20T-HA-*rpoD*-His was introduced into these strains.
